# 1-Methyl-3-phenyl­quinoxalin-2(1*H*)-one

**DOI:** 10.1107/S160053680903414X

**Published:** 2009-09-05

**Authors:** Hanane Benzeid, El Mokhtar Essassi, Nathalie Saffon, Bernard Garrigues, Seik Weng Ng

**Affiliations:** a Laboratoire de Chimie Organique Hétérocyclique, Pôle de compétences Pharmacochimie, Université Mohammed V-Agdal, BP 1014 Avenue Ibn Batout, Rabat, Morocco; b Service commun Rayons X, Université Paul Sabatier, Bâtiment 2R1, 118 route de Narbonne, 31062 Toulouse, France; c Hétérochimie Fondamentale et Appliquée, Université Paul Sabatier, UMR 5069, 118 Route de Narbonne, 31062 Toulouse, France; d Department of Chemistry, University of Malaya, 50603 Kuala Lumpur, Malaysia

## Abstract

The phenyl substituents in both independent mol­ecules of the title compound, C_15_H_12_N_2_O, are twisted with respect to the quinoxaline system [dihedral angles = 19.3 (1) and 30.4 (1)°].

## Related literature

For the structure of 1-ethyl-3-methyl­quinoxalin-2(1*H*)-one, see: Benzeid *et al.* (2008 ▶).
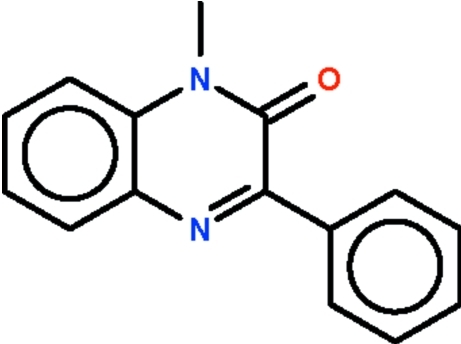


## Experimental

### 

#### Crystal data

C_15_H_12_N_2_O
*M* *_r_* = 236.27
Monoclinic, 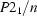

*a* = 16.3919 (5) Å
*b* = 7.0775 (2) Å
*c* = 20.0214 (6) Å
β = 95.434 (2)°
*V* = 2312.32 (12) Å^3^
*Z* = 8
Mo *K*α radiation
μ = 0.09 mm^−1^
*T* = 193 K
0.60 × 0.20 × 0.10 mm

#### Data collection

Bruker APEX2 diffractometer
Absorption correction: none
31360 measured reflections
4682 independent reflections
3068 reflections with *I* > 2σ(*I*)
*R*_int_ = 0.054

#### Refinement

*R*[*F* ^2^ > 2σ(*F* ^2^)] = 0.044
*wR*(*F* ^2^) = 0.114
*S* = 1.01
4682 reflections
327 parameters
H-atom parameters constrained
Δρ_max_ = 0.23 e Å^−3^
Δρ_min_ = −0.18 e Å^−3^

### 

Data collection: *APEX2* (Bruker, 2005 ▶); cell refinement: *SAINT* (Bruker, 2005 ▶); data reduction: *SAINT*; program(s) used to solve structure: *SHELXS97* (Sheldrick, 2008 ▶); program(s) used to refine structure: *SHELXL97* (Sheldrick, 2008 ▶); molecular graphics: *X-SEED* (Barbour, 2001 ▶); software used to prepare material for publication: *publCIF* (Westrip, 2009 ▶).

## Supplementary Material

Crystal structure: contains datablocks global, I. DOI: 10.1107/S160053680903414X/sj2638sup1.cif

Structure factors: contains datablocks I. DOI: 10.1107/S160053680903414X/sj2638Isup2.hkl

Additional supplementary materials:  crystallographic information; 3D view; checkCIF report

## Figures and Tables

**Fig. 1.**
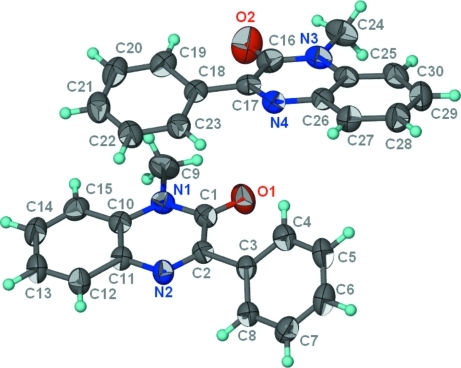
A thermal ellipsoid plot (Barbour, 2001) of the two unique molecules of C15H16N2O2 at the 70% probability level; hydrogen atoms are drawn as spheres of arbitrary radius.

## supplementary crystallographic information

### Experimental

3-Phenylquinoxalin-2(*1H*)-one (1 g, 4.5 mmol), methyl iodide (0.31 ml, 5.0 mmol), potassium carbonate (0.7 g, 5.0 mmol) and a catalytic quantity of tetra-*n*-butylammonium bromide were stirred in *N*,*N*-dimethylformamide (20 ml) for 24 h. The mixture was filtered to remove the salts and the solvent removed under reduce pressure. The residue was recrystallized in ethanol to afford the pure compound in 80% yield.The formulation was established by proton and carbon-13 NMR spectroscopy in CDCl_3_.

### Refinement

Carbon-bound H-atoms were placed in calculated positions (C—H 0.95 to 0.98 Å) and were included in the refinement in the riding model approximation, with *U*(H) set to 1.2–1.5*U*(C).

### Crystal data

**Table:**

C_15_H_12_N_2_O	*F*(000) = 992
*M**_r_* = 236.27	*D*_x_ = 1.357 Mg m^−^^3^
Monoclinic, *P*2_1_/*n*	Mo *K*α radiation, λ = 0.71073 Å
Hall symbol: -P 2yn	Cell parameters from 4338 reflections
*a* = 16.3919 (5) Å	θ = 2.5–24.9°
*b* = 7.0775 (2) Å	µ = 0.09 mm^−^^1^
*c* = 20.0214 (6) Å	*T* = 193 K
β = 95.434 (2)°	Plate, yellow
*V* = 2312.32 (12) Å^3^	0.60 × 0.20 × 0.10 mm
*Z* = 8	

### Data collection

**Table:**

Bruker APEX2 diffractometer	3068 reflections with *I* > 2σ(*I*)
Radiation source: fine-focus sealed tube	*R*_int_ = 0.054
graphite	θ_max_ = 26.4°, θ_min_ = 5.2°
φ and ω scans	*h* = −20→20
31360 measured reflections	*k* = −8→8
4682 independent reflections	*l* = −25→24

### Refinement

**Table:**

Refinement on *F*^2^	Primary atom site location: structure-invariant direct methods
Least-squares matrix: full	Secondary atom site location: difference Fourier map
*R*[*F*^2^ > 2σ(*F*^2^)] = 0.044	Hydrogen site location: inferred from neighbouring sites
*wR*(*F*^2^) = 0.114	H-atom parameters constrained
*S* = 1.00	*w* = 1/[σ^2^(*F*_o_^2^) + (0.0498*P*)^2^ + 0.4004*P*] where *P* = (*F*_o_^2^ + 2*F*_c_^2^)/3
4682 reflections	(Δ/σ)_max_ = 0.001
327 parameters	Δρ_max_ = 0.23 e Å^−^^3^
0 restraints	Δρ_min_ = −0.17 e Å^−^^3^

### Fractional atomic coordinates and isotropic or equivalent isotropic displacement parameters (Å^2^)

**Table:**

	*x*	*y*	*z*	*U*_iso_*/*U*_eq_	
O1	0.53417 (7)	0.34744 (19)	0.19948 (6)	0.0472 (4)	
O2	0.62711 (8)	0.8355 (2)	0.09382 (7)	0.0632 (4)	
N1	0.42018 (8)	0.3190 (2)	0.12599 (7)	0.0323 (3)	
N2	0.33162 (8)	0.2202 (2)	0.23283 (6)	0.0326 (3)	
N3	0.69753 (8)	0.8664 (2)	0.19620 (7)	0.0358 (4)	
N4	0.54955 (8)	0.86008 (19)	0.25385 (7)	0.0317 (3)	
C1	0.46011 (10)	0.3149 (2)	0.18965 (9)	0.0331 (4)	
C2	0.40856 (10)	0.2669 (2)	0.24408 (8)	0.0300 (4)	
C3	0.44373 (10)	0.2670 (2)	0.31523 (8)	0.0309 (4)	
C4	0.50477 (10)	0.3928 (3)	0.34009 (9)	0.0389 (4)	
H4	0.5281	0.4779	0.3105	0.047*	
C5	0.53143 (11)	0.3938 (3)	0.40790 (9)	0.0435 (5)	
H5	0.5728	0.4802	0.4246	0.052*	
C6	0.49836 (11)	0.2705 (3)	0.45122 (9)	0.0453 (5)	
H6	0.5169	0.2722	0.4976	0.054*	
C7	0.43825 (11)	0.1445 (3)	0.42723 (9)	0.0418 (5)	
H7	0.4157	0.0590	0.4571	0.050*	
C8	0.41091 (10)	0.1428 (2)	0.35977 (8)	0.0364 (4)	
H8	0.3694	0.0562	0.3435	0.044*	
C9	0.46906 (12)	0.3662 (3)	0.07079 (9)	0.0464 (5)	
H9A	0.4507	0.4876	0.0512	0.070*	
H9B	0.4622	0.2674	0.0364	0.070*	
H9C	0.5270	0.3751	0.0878	0.070*	
C10	0.33732 (10)	0.2762 (2)	0.11315 (8)	0.0306 (4)	
C11	0.29496 (10)	0.2221 (2)	0.16767 (8)	0.0309 (4)	
C12	0.21273 (10)	0.1659 (3)	0.15667 (9)	0.0382 (4)	
H12	0.1844	0.1259	0.1935	0.046*	
C13	0.17287 (11)	0.1683 (3)	0.09346 (9)	0.0428 (5)	
H13	0.1173	0.1291	0.0862	0.051*	
C14	0.21443 (12)	0.2287 (3)	0.03988 (9)	0.0438 (5)	
H14	0.1863	0.2334	−0.0038	0.053*	
C15	0.29569 (11)	0.2818 (2)	0.04900 (8)	0.0380 (4)	
H15	0.3232	0.3221	0.0118	0.046*	
C16	0.62597 (11)	0.8499 (3)	0.15482 (9)	0.0382 (4)	
C17	0.54917 (10)	0.8488 (2)	0.18917 (8)	0.0309 (4)	
C18	0.46772 (10)	0.8295 (2)	0.14974 (8)	0.0312 (4)	
C19	0.45321 (12)	0.8707 (3)	0.08144 (9)	0.0407 (4)	
H19	0.4969	0.9137	0.0573	0.049*	
C20	0.37535 (13)	0.8491 (3)	0.04856 (10)	0.0472 (5)	
H20	0.3661	0.8780	0.0021	0.057*	
C21	0.31130 (12)	0.7860 (3)	0.08274 (10)	0.0469 (5)	
H21	0.2584	0.7694	0.0597	0.056*	
C22	0.32428 (11)	0.7471 (3)	0.15040 (10)	0.0441 (5)	
H22	0.2802	0.7051	0.1742	0.053*	
C23	0.40153 (10)	0.7693 (2)	0.18359 (9)	0.0369 (4)	
H23	0.4098	0.7432	0.2303	0.044*	
C24	0.77511 (11)	0.8735 (3)	0.16470 (10)	0.0487 (5)	
H24A	0.7635	0.8699	0.1158	0.073*	
H24B	0.8091	0.7647	0.1795	0.073*	
H24C	0.8043	0.9905	0.1778	0.073*	
C25	0.69876 (10)	0.8736 (2)	0.26575 (9)	0.0341 (4)	
C26	0.62318 (10)	0.8714 (2)	0.29362 (8)	0.0314 (4)	
C27	0.62170 (11)	0.8786 (3)	0.36323 (9)	0.0402 (4)	
H27	0.5707	0.8792	0.3821	0.048*	
C28	0.69355 (12)	0.8848 (3)	0.40451 (10)	0.0479 (5)	
H28	0.6923	0.8890	0.4518	0.057*	
C29	0.76810 (12)	0.8850 (3)	0.37677 (10)	0.0499 (5)	
H29	0.8176	0.8885	0.4055	0.060*	
C30	0.77140 (11)	0.8802 (3)	0.30840 (10)	0.0437 (5)	
H30	0.8229	0.8813	0.2902	0.052*	

### Atomic displacement parameters (Å^2^)

**Table:**

	*U*^11^	*U*^22^	*U*^33^	*U*^12^	*U*^13^	*U*^23^
O1	0.0283 (7)	0.0654 (9)	0.0483 (8)	−0.0051 (6)	0.0063 (6)	−0.0005 (7)
O2	0.0531 (9)	0.1003 (13)	0.0387 (8)	0.0090 (8)	0.0178 (7)	−0.0003 (8)
N1	0.0342 (8)	0.0341 (8)	0.0294 (8)	−0.0007 (6)	0.0075 (6)	−0.0014 (6)
N2	0.0297 (8)	0.0389 (8)	0.0288 (8)	−0.0005 (7)	0.0010 (6)	−0.0027 (6)
N3	0.0315 (8)	0.0342 (8)	0.0439 (9)	0.0005 (7)	0.0150 (7)	−0.0008 (7)
N4	0.0310 (7)	0.0315 (8)	0.0330 (8)	0.0001 (6)	0.0052 (6)	−0.0016 (6)
C1	0.0288 (9)	0.0313 (9)	0.0396 (10)	0.0018 (7)	0.0047 (8)	−0.0034 (8)
C2	0.0278 (9)	0.0288 (9)	0.0334 (9)	0.0018 (7)	0.0023 (7)	−0.0033 (7)
C3	0.0241 (8)	0.0348 (9)	0.0334 (9)	0.0041 (7)	0.0007 (7)	−0.0022 (8)
C4	0.0324 (9)	0.0425 (11)	0.0415 (11)	0.0004 (8)	0.0018 (8)	−0.0024 (8)
C5	0.0340 (10)	0.0506 (12)	0.0439 (11)	0.0010 (9)	−0.0061 (8)	−0.0103 (9)
C6	0.0403 (11)	0.0583 (13)	0.0354 (10)	0.0099 (10)	−0.0061 (8)	−0.0055 (10)
C7	0.0387 (10)	0.0496 (12)	0.0366 (10)	0.0058 (9)	0.0004 (8)	0.0066 (9)
C8	0.0311 (9)	0.0396 (10)	0.0376 (10)	−0.0010 (8)	−0.0009 (8)	0.0008 (8)
C9	0.0509 (11)	0.0492 (12)	0.0417 (11)	−0.0096 (10)	0.0174 (9)	−0.0008 (9)
C10	0.0336 (9)	0.0266 (9)	0.0315 (9)	0.0030 (7)	0.0029 (7)	−0.0033 (7)
C11	0.0303 (9)	0.0321 (9)	0.0298 (9)	0.0032 (7)	0.0014 (7)	−0.0035 (7)
C12	0.0308 (9)	0.0485 (11)	0.0350 (10)	0.0004 (8)	0.0015 (8)	−0.0013 (8)
C13	0.0319 (10)	0.0520 (12)	0.0427 (11)	0.0014 (9)	−0.0059 (8)	−0.0030 (9)
C14	0.0457 (11)	0.0479 (12)	0.0353 (10)	0.0051 (9)	−0.0089 (9)	−0.0018 (9)
C15	0.0479 (11)	0.0362 (10)	0.0300 (9)	0.0010 (9)	0.0036 (8)	0.0008 (8)
C16	0.0401 (10)	0.0398 (11)	0.0361 (10)	0.0026 (8)	0.0110 (8)	0.0025 (8)
C17	0.0362 (9)	0.0241 (9)	0.0332 (10)	0.0025 (7)	0.0082 (7)	−0.0006 (7)
C18	0.0353 (9)	0.0259 (9)	0.0326 (9)	0.0056 (7)	0.0045 (7)	−0.0030 (7)
C19	0.0489 (11)	0.0371 (10)	0.0363 (10)	0.0060 (9)	0.0046 (9)	−0.0018 (8)
C20	0.0572 (13)	0.0453 (12)	0.0367 (10)	0.0127 (10)	−0.0077 (10)	−0.0053 (9)
C21	0.0431 (11)	0.0407 (11)	0.0538 (13)	0.0071 (9)	−0.0109 (10)	−0.0133 (9)
C22	0.0370 (10)	0.0414 (11)	0.0533 (12)	−0.0002 (9)	0.0004 (9)	−0.0040 (9)
C23	0.0359 (10)	0.0364 (10)	0.0381 (10)	0.0020 (8)	0.0017 (8)	−0.0015 (8)
C24	0.0368 (10)	0.0467 (12)	0.0666 (13)	−0.0015 (9)	0.0262 (10)	−0.0030 (10)
C25	0.0331 (9)	0.0252 (9)	0.0445 (10)	0.0003 (7)	0.0066 (8)	−0.0002 (8)
C26	0.0293 (9)	0.0280 (9)	0.0372 (10)	0.0016 (7)	0.0044 (7)	−0.0008 (7)
C27	0.0383 (10)	0.0439 (11)	0.0384 (10)	0.0012 (9)	0.0040 (8)	−0.0013 (8)
C28	0.0502 (12)	0.0499 (12)	0.0417 (11)	0.0009 (10)	−0.0058 (9)	−0.0030 (9)
C29	0.0405 (11)	0.0478 (12)	0.0582 (13)	0.0006 (9)	−0.0116 (10)	−0.0024 (10)
C30	0.0301 (10)	0.0380 (11)	0.0631 (13)	−0.0019 (8)	0.0049 (9)	−0.0008 (9)

### Geometric parameters (Å, °)

**Table:**

O1—C1	1.2331 (19)	C12—H12	0.9500
O2—C16	1.228 (2)	C13—C14	1.391 (3)
N1—C1	1.377 (2)	C13—H13	0.9500
N1—C10	1.392 (2)	C14—C15	1.380 (2)
N1—C9	1.464 (2)	C14—H14	0.9500
N2—C2	1.3027 (19)	C15—H15	0.9500
N2—C11	1.384 (2)	C16—C17	1.491 (2)
N3—C16	1.376 (2)	C17—C18	1.491 (2)
N3—C25	1.392 (2)	C18—C19	1.396 (2)
N3—C24	1.473 (2)	C18—C23	1.399 (2)
N4—C17	1.297 (2)	C19—C20	1.388 (3)
N4—C26	1.384 (2)	C19—H19	0.9500
C1—C2	1.480 (2)	C20—C21	1.381 (3)
C2—C3	1.485 (2)	C20—H20	0.9500
C3—C4	1.395 (2)	C21—C22	1.379 (3)
C3—C8	1.396 (2)	C21—H21	0.9500
C4—C5	1.386 (2)	C22—C23	1.382 (2)
C4—H4	0.9500	C22—H22	0.9500
C5—C6	1.377 (3)	C23—H23	0.9500
C5—H5	0.9500	C24—H24A	0.9800
C6—C7	1.381 (3)	C24—H24B	0.9800
C6—H6	0.9500	C24—H24C	0.9800
C7—C8	1.382 (2)	C25—C30	1.399 (2)
C7—H7	0.9500	C25—C26	1.406 (2)
C8—H8	0.9500	C26—C27	1.397 (2)
C9—H9A	0.9800	C27—C28	1.374 (2)
C9—H9B	0.9800	C27—H27	0.9500
C9—H9C	0.9800	C28—C29	1.389 (3)
C10—C15	1.397 (2)	C28—H28	0.9500
C10—C11	1.401 (2)	C29—C30	1.376 (3)
C11—C12	1.402 (2)	C29—H29	0.9500
C12—C13	1.368 (2)	C30—H30	0.9500
			
C1—N1—C10	122.44 (14)	C13—C14—H14	119.4
C1—N1—C9	117.23 (14)	C14—C15—C10	119.70 (17)
C10—N1—C9	120.31 (14)	C14—C15—H15	120.1
C2—N2—C11	119.18 (14)	C10—C15—H15	120.1
C16—N3—C25	122.45 (14)	O2—C16—N3	120.92 (16)
C16—N3—C24	117.81 (15)	O2—C16—C17	123.49 (16)
C25—N3—C24	119.73 (15)	N3—C16—C17	115.58 (15)
C17—N4—C26	119.98 (14)	N4—C17—C16	122.41 (15)
O1—C1—N1	121.22 (15)	N4—C17—C18	116.98 (15)
O1—C1—C2	123.17 (16)	C16—C17—C18	120.59 (15)
N1—C1—C2	115.60 (14)	C19—C18—C23	118.03 (16)
N2—C2—C1	122.79 (15)	C19—C18—C17	124.07 (16)
N2—C2—C3	116.58 (14)	C23—C18—C17	117.89 (15)
C1—C2—C3	120.62 (14)	C20—C19—C18	120.34 (18)
C4—C3—C8	118.73 (15)	C20—C19—H19	119.8
C4—C3—C2	122.86 (15)	C18—C19—H19	119.8
C8—C3—C2	118.31 (15)	C21—C20—C19	120.59 (18)
C5—C4—C3	120.11 (17)	C21—C20—H20	119.7
C5—C4—H4	119.9	C19—C20—H20	119.7
C3—C4—H4	119.9	C22—C21—C20	119.84 (18)
C6—C5—C4	120.47 (18)	C22—C21—H21	120.1
C6—C5—H5	119.8	C20—C21—H21	120.1
C4—C5—H5	119.8	C21—C22—C23	119.92 (19)
C5—C6—C7	120.04 (17)	C21—C22—H22	120.0
C5—C6—H6	120.0	C23—C22—H22	120.0
C7—C6—H6	120.0	C22—C23—C18	121.26 (17)
C6—C7—C8	120.00 (18)	C22—C23—H23	119.4
C6—C7—H7	120.0	C18—C23—H23	119.4
C8—C7—H7	120.0	N3—C24—H24A	109.5
C7—C8—C3	120.65 (16)	N3—C24—H24B	109.5
C7—C8—H8	119.7	H24A—C24—H24B	109.5
C3—C8—H8	119.7	N3—C24—H24C	109.5
N1—C9—H9A	109.5	H24A—C24—H24C	109.5
N1—C9—H9B	109.5	H24B—C24—H24C	109.5
H9A—C9—H9B	109.5	N3—C25—C30	122.88 (16)
N1—C9—H9C	109.5	N3—C25—C26	117.83 (15)
H9A—C9—H9C	109.5	C30—C25—C26	119.28 (17)
H9B—C9—H9C	109.5	N4—C26—C27	118.65 (15)
N1—C10—C15	123.16 (15)	N4—C26—C25	121.70 (15)
N1—C10—C11	117.56 (14)	C27—C26—C25	119.65 (16)
C15—C10—C11	119.28 (16)	C28—C27—C26	120.42 (17)
N2—C11—C10	122.27 (15)	C28—C27—H27	119.8
N2—C11—C12	118.07 (15)	C26—C27—H27	119.8
C10—C11—C12	119.66 (15)	C27—C28—C29	119.73 (18)
C13—C12—C11	120.63 (17)	C27—C28—H28	120.1
C13—C12—H12	119.7	C29—C28—H28	120.1
C11—C12—H12	119.7	C30—C29—C28	121.12 (18)
C12—C13—C14	119.40 (17)	C30—C29—H29	119.4
C12—C13—H13	120.3	C28—C29—H29	119.4
C14—C13—H13	120.3	C29—C30—C25	119.79 (17)
C15—C14—C13	121.25 (17)	C29—C30—H30	120.1
C15—C14—H14	119.4	C25—C30—H30	120.1
			
C10—N1—C1—O1	177.65 (15)	C25—N3—C16—O2	−176.73 (17)
C9—N1—C1—O1	−0.8 (2)	C24—N3—C16—O2	2.3 (3)
C10—N1—C1—C2	−1.6 (2)	C25—N3—C16—C17	2.6 (2)
C9—N1—C1—C2	179.90 (15)	C24—N3—C16—C17	−178.34 (15)
C11—N2—C2—C1	−2.6 (2)	C26—N4—C17—C16	−0.8 (2)
C11—N2—C2—C3	178.43 (14)	C26—N4—C17—C18	177.68 (14)
O1—C1—C2—N2	−175.20 (16)	O2—C16—C17—N4	178.43 (17)
N1—C1—C2—N2	4.1 (2)	N3—C16—C17—N4	−0.9 (2)
O1—C1—C2—C3	3.7 (3)	O2—C16—C17—C18	0.0 (3)
N1—C1—C2—C3	−177.04 (14)	N3—C16—C17—C18	−179.32 (14)
N2—C2—C3—C4	−147.10 (16)	N4—C17—C18—C19	160.36 (16)
C1—C2—C3—C4	33.9 (2)	C16—C17—C18—C19	−21.2 (2)
N2—C2—C3—C8	29.2 (2)	N4—C17—C18—C23	−18.6 (2)
C1—C2—C3—C8	−149.75 (16)	C16—C17—C18—C23	159.91 (15)
C8—C3—C4—C5	−0.4 (3)	C23—C18—C19—C20	−0.9 (2)
C2—C3—C4—C5	175.90 (16)	C17—C18—C19—C20	−179.88 (16)
C3—C4—C5—C6	0.3 (3)	C18—C19—C20—C21	−0.3 (3)
C4—C5—C6—C7	0.1 (3)	C19—C20—C21—C22	1.2 (3)
C5—C6—C7—C8	−0.4 (3)	C20—C21—C22—C23	−0.8 (3)
C6—C7—C8—C3	0.3 (3)	C21—C22—C23—C18	−0.5 (3)
C4—C3—C8—C7	0.1 (3)	C19—C18—C23—C22	1.3 (3)
C2—C3—C8—C7	−176.37 (15)	C17—C18—C23—C22	−179.67 (15)
C1—N1—C10—C15	179.25 (15)	C16—N3—C25—C30	176.49 (16)
C9—N1—C10—C15	−2.3 (2)	C24—N3—C25—C30	−2.5 (2)
C1—N1—C10—C11	−1.9 (2)	C16—N3—C25—C26	−2.6 (2)
C9—N1—C10—C11	176.53 (15)	C24—N3—C25—C26	178.38 (15)
C2—N2—C11—C10	−1.3 (2)	C17—N4—C26—C27	−178.43 (16)
C2—N2—C11—C12	178.21 (15)	C17—N4—C26—C25	0.9 (2)
N1—C10—C11—N2	3.5 (2)	N3—C25—C26—N4	0.8 (2)
C15—C10—C11—N2	−177.55 (15)	C30—C25—C26—N4	−178.34 (15)
N1—C10—C11—C12	−175.94 (15)	N3—C25—C26—C27	−179.93 (16)
C15—C10—C11—C12	3.0 (2)	C30—C25—C26—C27	0.9 (2)
N2—C11—C12—C13	178.77 (16)	N4—C26—C27—C28	178.28 (16)
C10—C11—C12—C13	−1.7 (3)	C25—C26—C27—C28	−1.0 (3)
C11—C12—C13—C14	−0.6 (3)	C26—C27—C28—C29	0.4 (3)
C12—C13—C14—C15	1.6 (3)	C27—C28—C29—C30	0.4 (3)
C13—C14—C15—C10	−0.3 (3)	C28—C29—C30—C25	−0.5 (3)
N1—C10—C15—C14	176.88 (16)	N3—C25—C30—C29	−179.29 (17)
C11—C10—C15—C14	−2.0 (2)	C26—C25—C30—C29	−0.2 (3)
